# Fluidic Grooves on Doped-Ice Surface as Size-Tunable Channels

**DOI:** 10.1038/srep17308

**Published:** 2015-11-25

**Authors:** Arinori Inagawa, Makoto Harada, Tetsuo Okada

**Affiliations:** 1 Department of Chemistry, Tokyo Institute of Technology, Meguro-ku, Tokyo 152-8551, Japan

## Abstract

We propose a new principle for fabrication of size-tunable fluidic nano- and microchannels with a ubiquitous green material, water. Grooves filled with a solution are spontaneously formed on the surface of ice when an appropriate dopant is incorporated. Sucrose doping allows the development of grooves with lengths of 300 μm along the boundaries of ice crystal grains. This paper focuses on controlling the size of the liquid-filled groove and reveals its applicability to size-selective differentiation of nano- and micromaterials. The width of this groove can be varied in a range of 200 nm to 4 μm by adjusting the working temperature of the frozen platform. The channel dimension is reproducible as long as the same frozen condition is employed. We demonstrate the size-selective entrapment of particles as well as the state evaluation of DNA by controlling the physical interference of the ice wall with the electrophoretic migration of particles.

Nano- and microfluidic channel devices are currently recognized as common platforms for reaction and separation of a wide variety of materials, including small molecules, macromolecules, particles, and biological cells. Micromachining techniques have allowed the fabrication of nano- and microfluidic channels with dimensions as low as a few tens of nanometers^1^,^2^. Small dimensions are expected to elicit novel fluidic aspects that would be of physical or chemical interest^3^,^4^,^5^. Channel walls play a more critical role in nano- and microspaces than in bulk solutions. A number of unusual phenomena, such as the overlapped electrical double layer^6^,^7^, viscosity change^8^, and slip flow at the wall^9^,^10^, have been known to be those coming from the wall effects. Some of these properties have been successfully utilized in separation or detection systems. A slip flow, for example, resulted in high separation performance in protein chromatography^11^.

Channel dimensions should be carefully designed to satisfy experimental requirements because it is difficult to change the size of a channel prepared on typical solid or polymer platforms^12^. Whereas modification of the sizes of nanopores with external stimuli, including pH, electrolyte concentration, and temperature, has been proposed^13^,^14^,^15^, effective methods for size tuning of larger-scale channels after their fabrication are still limited. Huh *et al.*^16^ fabricated a polydimethylsiloxane (PDMS) channel with a triangular cross-section, the size of which was varied by altering the compressive stress. Because of the elastomeric nature of PDMS, the channel shrank under high compressive stress and relaxed as the stress was removed. Nanoparticle separation from a dye solution and DNA manipulation were attempted by tuning the size of the channel. However, fine tuning is difficult with application of compressive stress on PDMS of high mechanical flexibility. Haulot *et al.*^17^ devised an optoelectronic reconfigurable microchannel device, in which the size of a channel fabricated on a frozen thin layer was controlled by optoelectronic heating. Fluidic channels with a size of a few hundreds of micrometers were constructed mainly on a frozen thin layer of dioxane, water, cyclohexane, or hexadecane, and their sizes and shapes were successfully controlled by the optoelectronic device.

In this paper, we propose a new concept for fabrication of size-tunable nano- and microchannels using a ubiquitous green material, water. The channel is spontaneously formed by freezing an aqueous solution. The channel size can be varied simply by changing the working temperature. The principle of the channel size tunability is based on the thermodynamic nature of a eutectic mixture. When an aqueous solution of a salt or a sugar is frozen, the dopant is expelled from the ice crystals and is accumulated inside the ice grain boundaries (IGBs). At a temperature above the eutectic point of the system (*T*_eu_), the dopant is dissolved in the aqueous liquid phase (LP). The LP thus coexists with ice in the temperature range between *T*_eu_ and the melting point under a given condition. We have previously shown that a frozen solution is a useful platform for designing separation, reaction, and sample pretreatment systems^18^,^19^,^20^,^21^,^22^,^23^,^24^. In addition, freezing accelerates some reactions in the LP because of the freeze concentration^25^ and possibly ice-confinement^26^. An important feature of a frozen solution is that the LP volume can be precisely controlled by changing the temperature and dopant concentration^22^. A decrease in the temperature, for example, leads to a decrease in the LP volume, which can be quantitatively explained on the basis of the phase diagram of the system.

Controlling the channel dimensions with temperature would be possible even with a typical solid material. However, the thermal expansion of a solid material is, in general, very small, e.g., coefficients of volume expansion are on the order of 10^−4^–10^−5 ^K^−1^ for typical solid materials^27^. A temperature change of 10 K induces a change of only100–1000 ppm in the volume of such solid materials. In contrast, the same temperature change can lead to a ~10% change in the volume of the ice phase in a frozen aqueous solution. This suggests that the dimensions of fluidic channels in a frozen aqueous solution can be varied in a much wider range than those of a channel fabricated with typical solid materials.

Freezing is a stochastic process, which makes reproducible preparation of fluidic channels difficult. However, the shape and size of ice grains can be controlled in appropriate freezing conditions; this can be used to control the channel dimension as well. Here we demonstrate the fabrication of fluidic grooves with widths of 200 nm to 4 μm by utilizing the nature of ice crystallization from a solution containing an appropriate dopant, and their application to size-selective entrapment of nano- and micromaterials, including particles and a DNA molecule.

## Results and Discussion

### Morphology of surface IGBs

The morphology of IGBs as well as that of ice grains have been studied by various methods, including scanning electron microscopy (SEM)^28^, magnetic resonance imaging (MRI)^29^, and fluorescence microscopy. Although MRI probes the interior structure of an IGB in frozen samples well, the resolution is limited to submillimeter order and this method has no surface selectivity. Cryo-SEM is, in general, a powerful tool for probing surface morphologies, but the substantial sublimation of ice hinders the visualization of the real surface. A recent study of environmental SEM^30^ has allowed imaging of ice and IGBs at a moderately reduced pressure of 100 kPa. For selective visualization of surface IGBs, incorporation of a heavy element such as uranium is required. If a water-soluble fluorescent dye is incorporated in doped ice, it is selectively dissolved in the LP and emits fluorescence. This phenomenon allows selective visualization of the IGBs on the ice surface. Although the spatial resolution of a typical optical microscope is limited to a submicrometer range, this method is suitable for the selective visualization of surface IGBs.

**Figure 1:**
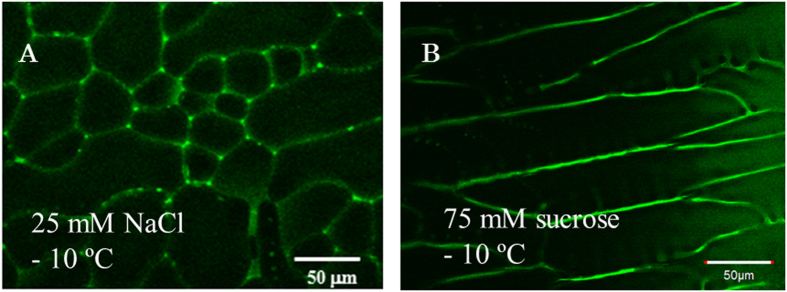
IGB channels formed on frozen NaCl (A) and frozen sucrose (B). Before freezing, the solutions contained 1.0 μm fluorescein disodium. The green and black areas represent the LP and ice, respectively.

Figure 1 shows confocal fluorescence microscopic images of NaCl- and sucrose-doped ice surfaces. Fluorescein was added together with the main dopant to selectively visualize the LP^20^. The green and dark parts in the figure represent the LP and ice grains, respectively. The images reveal that the surface morphology of the IGBs is strongly dependent on the nature of the main dopant. Most of the IGBs on the surface of salt-doped ice are almost hexagonally arranged, reflecting the crystal shape of ice Ih; the typical side length of a hexagonal ice crystal is 100–200 μm. In contrast, longer IGBs are arranged in parallel directions at approximately 50 μm intervals on the surface of sucrose-doped ice. Figure S1 shows additional examples of the surface images of sucrose-doped ice frozen at −6.0 °C. Interestingly, the IGB morphologies of the surface of sucrose-doped ice have similar characteristics in any preparation. These images indicate that an ice grain has a rectangular shape with a typical size of 50 μm × 300 μm. The IGB morphologies showed a small dependence on the freezing temperature. In this study, a frozen platform was prepared at −6.0 °C. However, freezing at a higher temperature resulted in larger IGB intervals, whereas a lower temperature caused smaller IGB intervals. The surface images obtained for freezing temperature at −4.0 °C and −10.0 °C are given in Figure S2. Because the ice crystal growth is faster at the lower temperature, the size of an ice grain becomes smaller as the freezing temperature is lowered. This causes smaller spaces between the IGBs on the surface prepared at the lower freezing temperature. In addition, the crystal growth direction becomes random at the lower freezing temperature because of the fast growth of ice crystals.

In this study, the electrophoretic behavior of nano- and micromaterials in long IGBs was determined. Measurements of electrophoretic migration rate were carried out when the long IGBs ran almost parallel to the line connecting two Ag/AgCl electrodes; otherwise, the ice platform was reconstructed. The parallel development of ice crystals in one direction in frozen sucrose suggests the dendritic growth of the crystal^31^,^32^,^33^.

**Figure 2:**
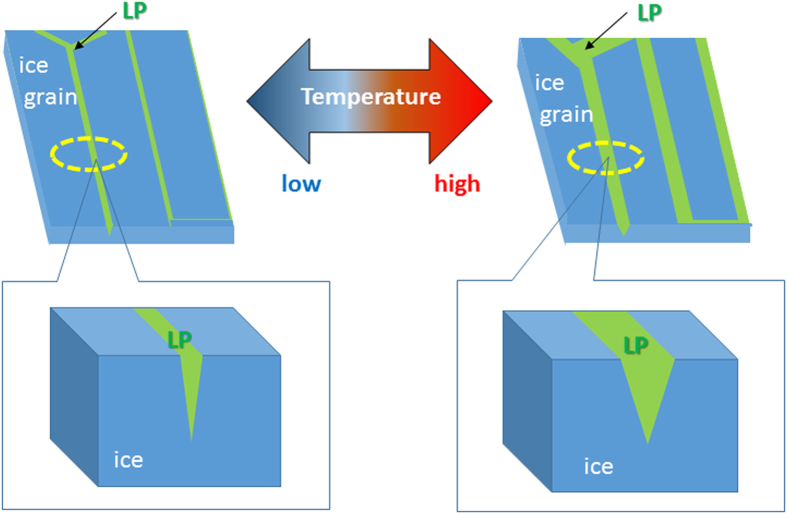
Schematic representation of IGB channel formation on the surface of ice and the control of channel size though temperature variation.

Figure S3 depicts a three-dimensional confocal fluorescence micrograph of the IGBs on the surface of 75 mM sucrose-doped ice. Although the precise size of the IGBs cannot be measured from this image because of vague interfaces due to similar refractive indexes of the LP and ice, the IGBs are formed between two ice plates arranged almost parallel to each other and have cross-sectional dimensions of approximately 20 μm depth and 1 μm width in this condition. The channel length is a few hundreds of micrometers as noted previously. The present concept of channel size tuning is illustrated in Fig. 2. Once ice crystals are formed, the grain size hardly changes with temperature because the channel length is determined by the grain size, which is independent of the working temperature. Contrarily, the channel cross-sectional area changes with temperature; an increase in the temperature causes an increase in the LP volume and in the channel cross-section.

### IGB electrophoresis of particles

**Figure 3:**
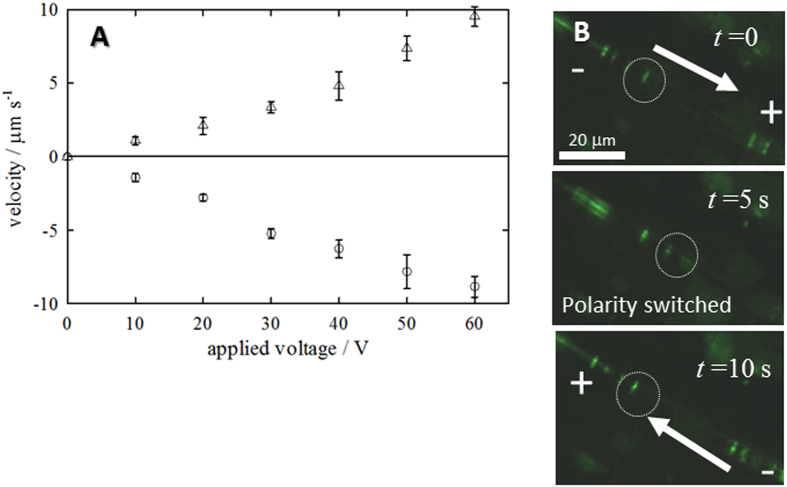
(**A**) Relations between applied voltage and the electrophoretic rate of negatively (triangles) and positively charged (circles) particles with *d* = 1.3 μm at −2.0 °C. Migration towards the anode was taken as positive. The measurements were triplicated (*n* = 3). (**B**) Images for the migration of the negatively charged particle with *d* = 1.3 μm in the IGB with a voltage of 63.1 V. The particle within the circle migrated toward the anode at *t* = 0. When the polarity was switched at *t* = 5 s, the direction of its migration was reversed.

Figure 3A shows the dependence of the electrophoretic velocity of a *d* = 1.3 μm particle (*d* is diameter) in the IGB channel on the applied voltage. The negatively charged particle migrated toward the anode, while the positively charged one migrated in the opposite direction. Moreover, the migration velocity of either particle was almost proportional to the applied voltage. The electrophoretic migration of the negatively charged particles along the IGB channel prepared with 75 mM sucrose at −2.0 °C is depicted in Fig. 3B. When the polarity of the applied voltage was switched, the particles started to migrate in the opposite direction. Thus, particles electrophoretically migrate in the IGB channel.

From the phase diagram of the water–sucrose system (Figure S4)^34^, the concentration of sucrose in the LP can be estimated at given temperatures. Although the matrix contains NaCl, its concentration is one-hundredth of that of sucrose. The system is therefore regarded as a binary sucrose–water mixture that follows the phase diagram illustrated in Figure S4. NaCl is therefore concentrated in the LP according to the ratio of the sucrose concentration in the LP to that in the original solution before freezing.

The electrical conductivity of the solution of the LP composition was measured as listed in Table S1. From these values and the current measured during electrophoretic runs, we can estimate the effective cross-sectional area of the channel between two probe electrodes. The values are also listed in Table S1. The effective cross-section is much larger than that expected from a single conductive path estimated from Figure S3. This strongly suggests the presence of multi-conductive paths between the electrodes. In reality, the migration rate of a given particle depended on the preparation of ice, indicating that electric field strength was varied for ice preparations. The nonlinearity seen in Fig. 1A can come from this complex conductive path in an ice matrix.

**Figure 4:**
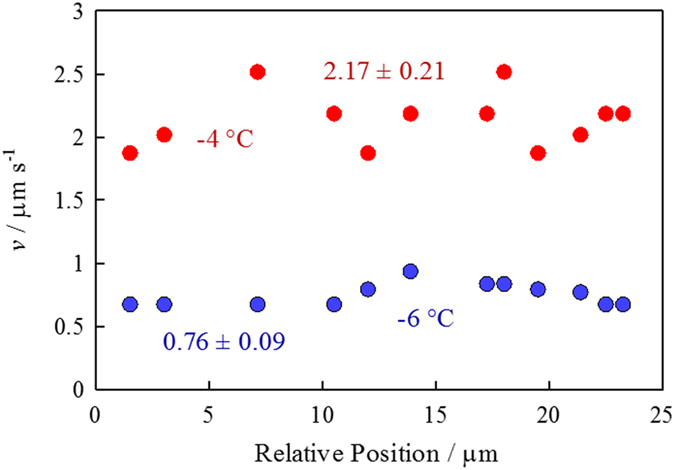
Variation of electrophoretic migration rate for *d* = 1.3 μm particles along a single IGB at −4.0 and −6.0 °C. The average migration rates and standard deviations at these temperatures are shown in the figure.

Figure 4 illustrates the local migration rates of the *d* = 1.3 μm particle along a single IGB at two different temperatures. The migration rate varies depending on the location along the IGB, suggesting that the IGB channel width is not uniform. The relative standard deviation of electrophoretic migration rates measured along an IGB is typically 10%. Therefore, although the channel dimension is not uniform in a rigorous sense, its heterogeneity is not very large and is at least smaller than the variation in the size of the particles; the relative standard deviation of particle diameter was 20% for all particle samples (see Method for details).

**Figure 5:**
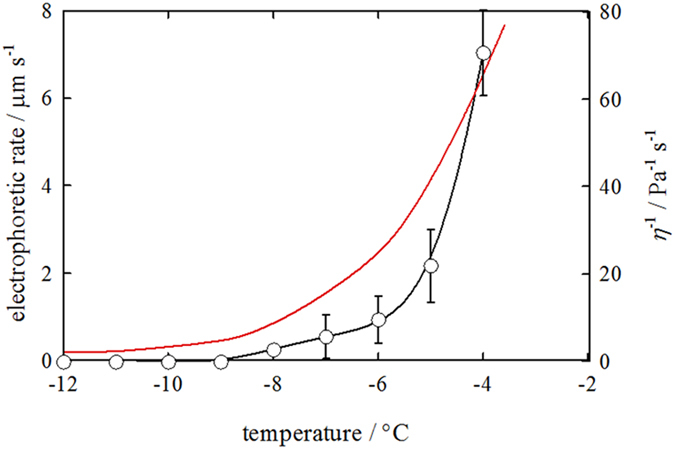
Temperature dependence of the migration rate for the *d* = 1.3 μm particle in an IGB channel prepared with *c*_suc_ = 75 mM. The prediction based on Eq. 1 is shown as a red curve. The viscosity of the LP was estimated as listed in Table S1. The measurements were triplicated (*n* = 3).

Figure S5 shows a comparison of the electrophoretic behavior of the *d* = 1.3 μm particle at −6.0 °C with that at −12.0 °C. Migration can be seen at −6.0 °C, whereas the particle is immobile at −12.0 °C. The migration rate was measured by changing the temperature at 1.0 °C intervals to reveal the temperature dependence of particle migration rate. Figure 5 summarizes the results obtained for the negatively charged *d* = 1.3 μm particle in the IGB prepared with 75 mM sucrose. The migration rate decreases with decreasing temperature, and the particle becomes immobile when the temperature decreases to below −9 °C. Although the migration of only one particle was measured considering the variations in particle sizes and uniformity of the channel width along an IGB, all particles in a microscopic view behaved in a similar manner. Several particles are often accommodated in the same IGB, and in some cases, a few IGB channels are simultaneously observed in a single microscopic view. When a particle under study becomes immobile with decreasing temperature, the migration of all other particles in a microscopic view also stops. Similarly, when the temperature increases, all particles start to migrate at the same temperature.

The migration velocity of a particle (*v*) is described by




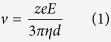




where *z* is the charge of a particle, *e* is the elementary charge, *E* is an electric field, and *η* is the viscosity of an electrophoretic medium. The viscosity of the LP depends on the solute concentration as well as the temperature. In the present case, both the sucrose concentration and the viscosity of the LP increase as the temperature decreases. The following equation explains the temperature dependence of the viscosity of the LP^35^,




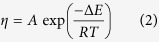




where Δ*E* is the flow activation energy. The viscosities of the LP in the present system at various temperatures were estimated with Eq. (2). The viscosity data for aqueous sucrose are listed in Table S2. The estimation procedure of the viscosity of the LP is described in detail in the Supplementary Information, and the determined parameters are summarized in Table S3. The viscosity data predicted for the LP at given temperatures are summarized in Table S4.

The electro-osmotic flow (EOF) should also contribute to the migration of particles in the IGB. Because EOF is proportional to 1/*η*, similar to electrophoretic rates, the total migration rate of a particle along the IGB is proportional to 1/*η*. The temperature dependence of 1/*η* of the LP, which is calculated by Eq. (2), is depicted as the red curve shown in Fig. 5. The actual effect of temperature on the electrophoretic rate is more marked than the prediction given by Eq. (1), suggesting that factors other than viscosity are involved in the effect of temperature on particle migration in the IGB channel. The viscosity predicted for the LP involves some ambiguities coming from the data in ambient conditions and regression analyses. However, as described in the following, smaller particles remain mobile at the temperature at which *d* = 1.3 μm particles are immobile. Factors other than viscosity should be responsible for the temperature dependence of particle migration; one possible factor is the physical interference of the ice wall with the particle movement.

**Figure 6:**
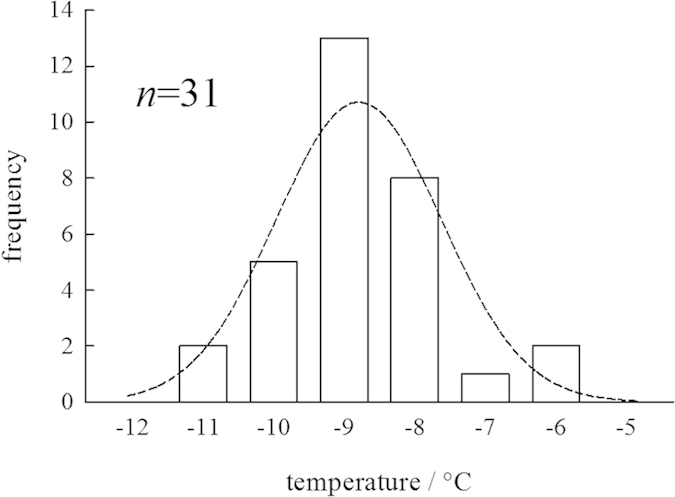
Repeated measurements of the threshold temperature for the *d* = 1.3 μm particle in the IGB channel prepared with 75 mM sucrose. The broken curve represents a Gaussian distribution with an average temperature of −8.8 °C and a standard deviation of 1.1 °C.

The effective electric field acting on particles was varied for each frozen sample preparation because of the inherently varying electric resistance of an ice platform as discussed previously. Therefore, the electrophoretic mobility of any given particle in the IGB showed variations, even in the same condition. Nevertheless, the threshold temperature, at which a given particle becomes immobile upon decreasing temperature, can be explicitly defined for a given dopant concentration. Repeated measurements of the threshold temperature for the *d* = 1.3 μm particle in the IGB prepared with 75 mM sucrose are summarized in Fig. 6. The threshold temperature ranged from −6.0 °C to −11.0 °C. Because freezing is a stochastic process, the distribution of the threshold temperature follows the Gaussian function. The average threshold temperature is −8.8 °C with *σ* = 1.1 °C. These results strongly suggest that the effective width of the IGB channel prepared with 75 mM sucrose becomes as small as 1.3 μm at this average temperature. The temperature dependence of particle migration showed no thermal hysteresis; particles entrapped in the IGB channel moved again when the temperature increased past the threshold temperature. Therefore, the particle behavior in the IGB channel can be controlled by altering the temperature.

The fluidity and flowability of the LP become poor at low temperatures because of increased viscosity of the LP as discussed previously. This will be a serious problem if pressurized flow is utilized in the IGB for the transport of materials through the channel. However, the effects of high viscosity of the LP on the electrophoretic transport over a short distance are not very notable.

**Figure 7:**
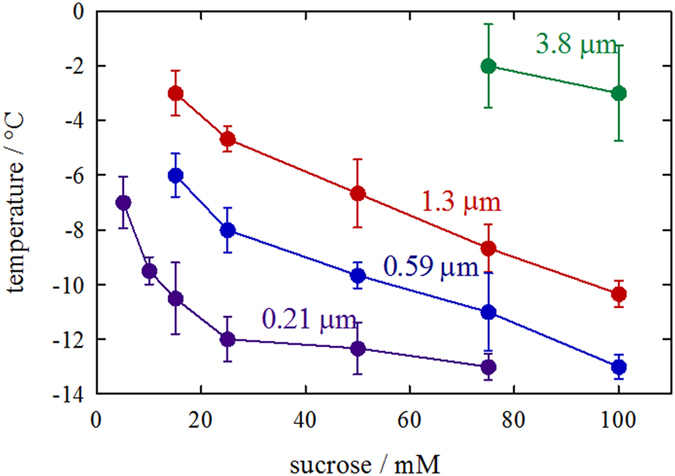
Changes in the threshold temperature with the *c*_suc_ used for frozen matrix preparation for four different particles. The temperature, at which a given particle became immobile when the temperature decreased, was defined as the threshold temperature. The threshold was confirmed by repeated increases and decreases in the temperature around this point. The determination of the threshold temperature was repeated three times on the independently prepared ice platform for most of the measurements; more measurements (five times or more) were performed for the 1.3 μm particle. The standard deviations were calculated based on the number of measurements for individual points. This figure can be regarded as a contour plot of effective channel width on a *c*_suc_-temperature plane.

Physical interference with particle migration from the ice wall of the IGB channel can be utilized for the determination of effective channel width. The migration of particles with *d* = 3.8, 1.3, 0.59, and 0.21 μm was examined using the IGB channel prepared by varying the sucrose concentration (*c*_suc_ = 5–100 mM). In Fig. 7, the threshold temperatures determined for these particles are plotted against *c*_suc_. This figure also presents the contour plot of effective IGB channel width on a *c*_suc_-temperature plane. This figure demonstrates that the effective channel width can be varied between 210 nm and 3.8 μm by controlling the temperature and *c*_suc._ The controllable channel width becomes smaller as *c*_suc_ decreases.

**Figure 8:**
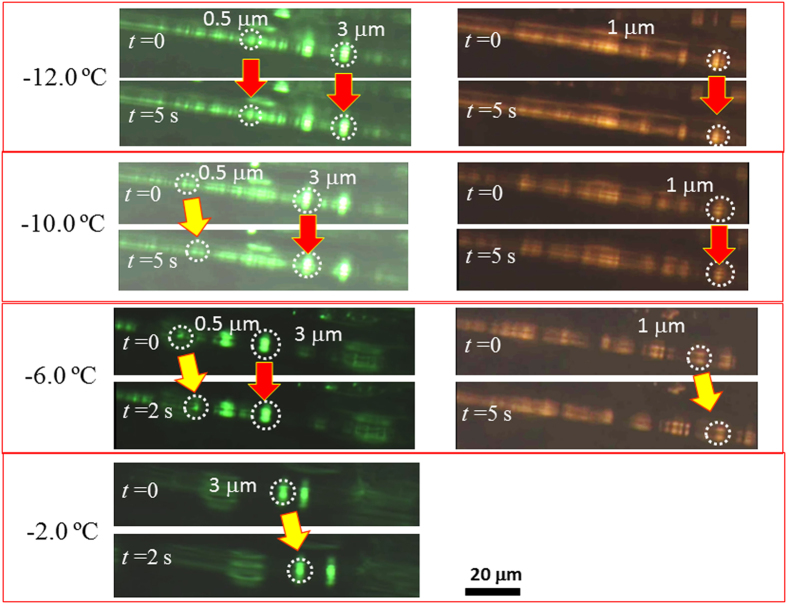
Electrophoretic separation of 0.59, 1.3, and 3.8 μm particles by IGB channel electrophoresis. Particles of three different sizes were simultaneously introduced in the channel. To discriminate individual migration, the 1.3 μm particle was stained with a different dye. Red arrows indicate that no migration was detected, while yellow arrows indicate the movement of the particles.

Figure 7 also gives a basis for the size differentiation of particles using the size-tunability of the IGB channel. For selective entrapment of micro-particles, a *c*_suc_ range of 75–100 mM will be appropriate, while a lower *c*_suc_ is suitable for smaller particles. The electrophoretic size resolution of particles is usually difficult in free solutions^36^,^37^. In order to overcome this problem, nanostructures were constructed in microchannels, or polymers, which provided sieving effects, were employed in running buffers to differentiate the migration rates of different-sized particles^38^,^39^. The IGB provides a concept entirely different from these known approaches. The selective entrapment of particles larger than a particular size or the selective retardation of their migration is feasible by utilizing temperature-controlled physical interference from the ice wall. Particles with *d* = 3.8, 1.3, and 0.59 μm were simultaneously introduced in the IGB channel prepared with *c*_suc_ = 75 mM, and their behaviors therein were observed at different temperatures. As discussed above, the effective width of the channel is 0.6, 1.3, and 3.8 μm at −11, −9, and −2.0 °C, respectively. Selected images are shown in Fig. 8. All of the particles electrophoretically travel along the IGB channel at −2.0 °C. However, when the temperature decreases to −6.0 °C, the migration of 3 μm particles ceases, whereas particles of other sizes remain mobile. The *d* = 1.3 μm particles become immobile at −10.0 °C, while the *d* = 0.59 μm particles still migrated at this temperature; at −12.0 °C, no particles migrate. The temperature-controlled size-separation based on channel width tuning has thus been successfully demonstrated.

When several particles are accommodated in a channel, entrapped larger particles may interrupt the electrophoretic migration of smaller particles because of clogging. However, as depicted in Figure S3, the channel depth is in the range of tens of micrometer. This suggests that smaller particles can migrate through the spaces above or below entrapped larger particles. Figure S6 shows examples of such situations, where 0.59 μm particles migrate through the already entrapped 3.8 μm particles. Thus, channel blocking does not occur even when samples of different sizes are introduced in an IGB channel.

### Potential application to biomaterials

**Figure 9:**
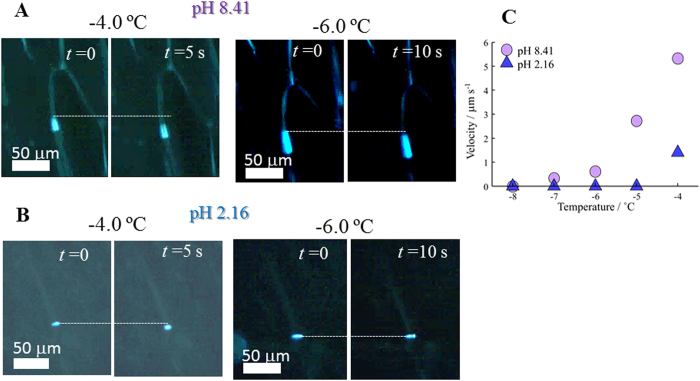
IGB channel electrophoresis of DNA. (**A**) Random-coil DNA at pH 8.41. (**B**) Globule DNA at pH 2.16. (**C**) Temperature dependence of electrophoretic migration rate for globule (pH 2.16) and random-coil DNA (pH 8.41).

The present concept is also applicable to biomaterials. The sizes of biological cells and some biopolymers are comparable to the width of the IGB channel; therefore, their migration in the channel should be affected by physical interference from the ice wall. Some images obtained for a gigantic DNA molecule (T4 GT7 DNA) are shown in Fig. 9. It is known that DNA changes its macrostructure through a coil-globule transition, which is induced by changes in pH or salt concentration^40^,^41^. Figure 9A shows that the DNA has a spheroidal shape at pH 8.41, and migrates through the IGB channel at both −4.0 and −6.0 °C. The DNA molecule adopts a coiled state at this pH and can fit its flexible contour to the channel shape, thereby reducing interference from the wall. However, the DNA looks like a solid particle at pH 2.16, and does not show electrophoretic movement below −4 °C (Fig. 9B). The DNA molecule adopts a globular state at pH 2.16 because of reduced electrostatic intramolecular repulsions. In this state, the DNA chain is packed into a small space and should have a rigid contour. Thus, it behaves like a solid particle, and its migration is effectively hindered because of physical interference from the wall. The temperature dependence of migration velocities in these two states is summarized in Fig. 9C. At pH 8.41, the DNA migrates above −7.0 °C, while, at pH 2.16, its migration is not detected at −5.0 °C. Thus, the size tunable nature of the IGB channel effectively characterizes the structural features of DNA.

## Conclusion

The size tunability of the IGB channel has been demonstrated with the temperature-controlled electrophoresis of particles and DNA. The minimum effective channel width that can be reproducibly prepared is currently ca 200 nm. One of the challenges of subsequent works will be the reduction of the minimum size, which will allow the application of the present concept to the size-selective separation of nanoparticles. The present method is not useful from a practical viewpoint because of low throughput. However, we believe that practical improvements to this method are feasible. For example, multiple channels fabricated in an ice septum can act as a size-tunable filter or sieve. Working on larger scales will prove the practical efficiency of the present method.

## Method

### Materials

Polystyrene particles (Polyscience Inc.) were used to evaluate the IGB channel width and to demonstrate its size tunability by changing the temperature and dopant concentration. The *d* = 0.21, 0.59, 1.3, and 3.8 μm particles were stained with Yellow Green (YG, λ_ex_ = 529 nm, λ_em_ = 546 nm), and the 1.3 μm particles stained with Yellow Orange (YO, λ_ex_ = 441 nm, λ_em_ = 486 nm) were also used. The particle diameters were evaluated by laser dynamic light scattering using a DLS-8000 from Photal. The average diameters of the *d* = 0.21, 0.59, 1.3, and 3.8 μm particles were 0.21 ± 0.04, 0.59 ± 0.11, 1.29 ± 0.26, and 3.82 ± 0.87 μm, respectively.

The behavior of T4 GT7 DNA (166 kbp, Nippon Gene) in the IGB was also studied. The DNA was dispersed in a TAE buffer containing 10 mM tris(hydroxymethyl)aminomethane (tris), 0.25 mM EDTA, and 5mM acetic acid adjusted to a pH of 2.16 or 8.41. DAPI (4′,6-diamino-2-phenylindole) solution was then added to the DNA solution to stain the DNA. The final concentrations of DNA and DAPI in the sample were 4.0 × 10^−13^ and 4.3 × 10^−7 ^M, respectively. Fluorescence from the stained DNA was measured with λ_ex_ = 330–385 nm and λ_em_ ≧420 nm. All solutions were prepared in Milli Q water. Reagents of analytical grade were used as received.

### Instruments

The experimental setup is depicted in Figure S7. The sample temperature was controlled using a Peltier unit, which was operated by a cell system Peltier controller (model: TDC-2030). The opposite side of the Peltier array was cooled by a chiller. The sample temperature was measured using a Pt resistance thermometer. The electrophoretic behavior of particles in the IGB channel was observed using an Olympus fluorescence microscope (model: BX41) with an Hg lamp light source or an Olympus confocal laser scanning microscope (model: FV1200); a ×50 objective (N/A = 0.5) was used. Videos were taken using a CCD camera (Shimadzu Moticam 2500). Electrophoretic voltage was applied using a DC voltage supplier (Kikusui Electronics Corp.). Two Ag-AgCl electrodes were fixed 2.0 mM apart in the Cu cell at a depth of 1.0 mM from the frozen sample surface. The side of the electrode was insulated with varnish, and only the tip was in contact with the frozen sample.

### IGB channel fabrication

The IGB channel was fabricated by simply freezing an aqueous solution. A sucrose solution containing NaCl, with a NaCl concentration one-hundredth of that of sucrose, was used as the matrix for channel fabrication. The TAE buffer was used as the matrix for the electrophoresis of DNA in the IGB. A sample solution was cooled in a hand-made copper cell pasted onto a Peltier unit (effective area = 6 cm × 6 cm). The inner wall of the cell was insulated with a coating of varnish.

The matrix solution (0.65 mL) was put in the Cu cell, which was cooled on the Peltier array set to −6.0 °C. Before the solution was completely frozen (when the thickness of the ice layer became ca 3 mm), a 15 μL aliquot of a sample (particles or DNA) prepared in the same solution as the matrix was added onto the partially frozen matrix. The concentrations of the particles were 5.68 × 10^9^, 3.64 × 10^8^, 4.55 × 10^7^, and 1.68 × 10^6 ^mL^−1^ for 0.21, 0.59, 1.3, and 3.8 μm particles, respectively After the solution was completely frozen, the temperature was lowered to −12.0 °C. The particles or DNAs were rejected from ice crystals and were spontaneously introduced into the IGBs.

The electrophoretic migration rates of the particles were measured in −12.0–−2.0 °C. After a constant temperature was reached, an electrophoretic voltage of 63.1 V was applied to measure the migration rate of the particles in the GB. Although a low voltage was preferable to avoid melting of ice due to joule heat, migration rate determination was difficult with a low electric field. The working voltage (63.1 V) was determined by taking these requirements into account. The migration rate was determined by measuring the migration distance for 0.5 s. One pixel on an image corresponded to 0.15 μm. When particle displacement was not detected over 10 s, the particle was considered immobile. The displacement was measured every 1.0 °C both while increasing the temperature from −12.0 to −2.0 °C and while decreasing it from −2.0 to −12.0 °C. The temperature at which a particle became immobile was defined as the threshold temperature.

## Additional Information

**How to cite this article**: Inagawa, A. *et al.* Fluidic Grooves on Doped-Ice Surface as Size-Tunable Channels. *Sci. Rep.* **5**, 17308; doi: 10.1038/srep17308 (2015).

## Supplementary Material

Supplementary Information

## References

[b1] Mijatovic D., Eijkel J. C. & van den Berg A. Technologies for Nanofluidic Systems: Top-down vs. Bottom-up–A Review. Lab on Chip 5, 492–500 (2005). 10.1039/b416951d 15856084

[b2] Gunther A. & Jensen K. F. Multiphase Microfluidics: from Flow Characteristics to Chemical and Materials Synthesis. Lab on Chip 6, 1487–1503 (2006). 10.1039/b609851g 17203152

[b3] Tsukahara T., Mawatari K. & Kitamori T. Integrated extended-Nano Chemical Systems on a Chip. Chem. Soc. Rev. 39, 1000–1013 (2010). 20179821 10.1039/b822557p

[b4] Mark D., Haeberle S., Roth G., von Stetten F. & Zengerle R. Microfluidic Lab-on-a-Chip Platforms: Requirements, Characteristics and Applications. Chem. Soc. Rev. 39, 1153–1182 (2010). 20179830 10.1039/b820557b

[b5] Ohno K., Tachikawa. K. & Manz A. Microfluidics: Applications for Analytical Purposes in Chemistry and Biochemistry. Electrophoresis 29, 4443–4453 (2008). 19035399 10.1002/elps.200800121

[b6] Huang K.-D. & Yang R.-J. Electrokinetic Behaviour of Overlapped Electric Double Layers in Nanofluidic Channels. Nanotechnol. 18, 115701 (2007).

[b7] Yaroshchuk A. E. Transport Properties of Long Straight Nano-Channels in Electrolyte Solutions: a Systematic Approach. Adv. Colloid Interface Sci. 168, 278–291 (2011). 21496786 10.1016/j.cis.2011.03.009

[b8] Hibara A. *et al.* Nanochannels on a Fused-Silica Microchip and Liquid Properties Investigation by Time-Resolved Fluorescence Measurements. Anal. Chem. 74, 6170–6176 (2002). 12510735 10.1021/ac025808b

[b9] Li L., Mo J. & Li Z. Flow and Slip Transition in Nanochannels. Phys. Rev. E 90, 033003 (2014). 10.1103/PhysRevE.90.033003 25314525

[b10] Tretheway D. C. & Meinhart C. D. Apparent Fluid Slip at Hydrophobic Microchannel Walls. Phys. Fluids 14, L9 (2002).

[b11] Wei B., Rogers B. J. & Wirth M. J. Slip Flow in Colloidal Crystals for Ultraefficient Chromatography. J. Am. Chem. Soc. 134, 10780–10782 (2012). 22708746 10.1021/ja304177m PMC3392167

[b12] Abgrall P. & Nguyen N. T. Nanofluidic Devices and Their Applications. Anal.Chem. 80, 2326–2341 (2008). 18321133 10.1021/ac702296u

[b13] Guo W. *et al.* Bio-inspired Two-dimensional Nanofluidic Generators Based on a Layered Graphene Hydrogel Membrane. Adv Mater 25, 6064–6068 (2013). 23900945 10.1002/adma.201302441

[b14] Guo W. *et al.* Target-specific 3D DNA Gatekeepers for Biomimetic Nanopores. Adv Mater 27, 2090–2095 (2015). 25683922 10.1002/adma.201405078

[b15] Guo W., Tian Y. & Jiang J. Asymmetric Ion Transport through Ion-Channel-Mimetic Solid-State Nanopores. Acc.Chem.Res. 46, 2834–2846 (2013). 23713693 10.1021/ar400024p

[b16] Huh D. *et al.* Tuneable Elastomeric Nanochannels for Nanofluidic Manipulation. Nat. Mater. 6, 424–428 (2007). 17486084 10.1038/nmat1907

[b17] Haulot G., Benahmed A. J. & Ho C. M. Optoelectronic Reconfigurable Microchannels. Lab on a chip 12, 5086–5092, (2012). 23081660 10.1039/c2lc40615b

[b18] Ito K. & Okada T. Freeze Sample Enrichment Highly Adaptable to Capillary Electrophoresis. Anal. Methods 5, 5912 (2013).

[b19] Okada T. Design of Analytical Systems Based on Functionality of Doped Ice. Anal.Sci. 30, 43–49 (2014). 24420243 10.2116/analsci.30.43

[b20] Hashimoto T., Tasaki Y., Harada M. & Okada T. Electrolyte-Doped Ice as a Platform for Atto- to Femtoliter Reactor Enabling Zeptomol Detection. Anal. Chem. 83, 3950–3956 (2011). 21469701 10.1021/ac200785n

[b21] Tasaki Y. & Okada T. Ice Chromatography. Characterization of Water-Ice as a Chromatographic Stationary Phase. Anal. Chem. 78, 4155–4160 (2006). 16771546 10.1021/ac0602470

[b22] Tasaki Y. & Okada T. Control of Ice Chromatographic Retention Mechanism by Changing Temperature and Dopant Concentration. Anal. Chem. 83, 9593–9599 (2011). 22053829 10.1021/ac202378m

[b23] Tasaki Y. & Okada T. Up to 4 Orders of Magnitude Enhancement of Crown Ether Complexation in an Aqueous Phase Coexistent with Ice. J. Am. Chem. Soc. 134, 6128–6131 (2012). 22468638 10.1021/ja301989d

[b24] Shamoto T., Tasaki Y. & Okada T. Chiral Ice Chromatography. J. Am. Chem. Soc. 132, 13135–13137 (2010). 20825182 10.1021/ja1055214

[b25] Takenaka N. & Bandow H. Chemical Kinetics of Reactions in the Unfrozen Solution of Ice. J. Phys. Chem.A 111, 8780–8786 (2007). 17705357 10.1021/jp0738356

[b26] Anzo K., Harada M. & Okada T. Enhanced Kinetics of Pseudo First-Order Hydrolysis in Liquid Phase Coexistent with Ice. J. Phys. Chem. A 117, 10619–10625 (2013). 24063609 10.1021/jp409126p

[b27] Ushiki S. in Kagaku Binran, (Chemical Index) 4th Edn.(ed. The Chemical Society of Japan) Ch.5, II-17 (Maruzen, 1993).

[b28] Blackford J. R. Sintering and Microstructure of Ice: a Review. Journal of Physics D: Applied Physics 40, R355–R385 (2007).

[b29] Galley R. J. *et al.* Morphology and Distribution of Liquid Inclusio ns in Young Sea Ice as Imaged by Magnetic Resonance. The Cryosphere Discussions 7, 4977–5006 (2013).

[b30] Krausko J., Runstuk J., Nedela V., Klan P. & Heger D. Observation of a Brine Layer on an Ice Surface with an Environmental Scanning Electron Microscope at Higher Pressures and Temperatures. Langmuir 30, 5441–5447, (2014). 24761934 10.1021/la500334e

[b31] Gonda T., Sei T. & Arima Y. The Morphology and the Growth Rate of Ice Crystals Growing in Aqueous Sucrose Solution. Bull. Glaciol. Res. 19, 13–17 (2002).

[b32] Uchida T., Nagayama M., Shibayama T. & Gohara K. Morphological Investigations of Disaccharide Molecules for Growth Inhibition of Ice Crystals. J. Cryst. Growth 299, 125–135, (2007).

[b33] Uchida T. & Takeya S. Powder X-ray Diffraction Observations of Ice Crystals Formed from Disaccharide Solutions. Phys. Chem. Chem. Phys. 12, 15034–15039 (2010). 20957238 10.1039/c0cp01059f

[b34] Young F. E. & Jones F. T. Sucrose Hydrates. The Sucrose–Water Phase Diagram. J. Phys. Chem. 53, 1334–1350 (1949).

[b35] Battezzati L. & Greer A. L. The Viscosity of Liquid Metals and Alloys. Acta Metall. 37, 1791–1802 (1989).

[b36] Radko S. P. & Chrambach A. Capillary Electrophoresis of Subcellular-Sized Particles. J. Chromatogr. B 722, 1–10 (1999). 10.1016/s0378-4347(98)00307-7 10068130

[b37] Radko S. P. & Chrambach A. Separation and Characterization of Sub-Micro-M- and Micro-M Sized Particles by Capillary Zone Electrophoresis. Electrophoresis 23, 1957–1972 (2002). 12210247 10.1002/1522-2683(200207)23:13<1957::AID-ELPS1957>3.0.CO;2-I

[b38] Chrambach A. & Aldroubi A. Relative Efficiency of Molecular Sieving in Solutions of Four Polymers. Electrophoresis 14, 18–22 (1993). 8462511 10.1002/elps.1150140104

[b39] Song X., Li L., Qian H., Fang N. & Ren J. Highly Efficient Size Separation of CdTe Quantum Dots by Capillary Gel Electrophoresis Using Polymer Solution as Sieving Medium. Electrophoresis 27, 1341–1346 (2006). 16502461 10.1002/elps.200500428

[b40] Inoshita S., Tsukahara S. & Fujiwara T. *In Situ* Fluorescence Microscopic Investigation into the Dependence of Conformation and Electrophoretic Velocity of Single DNA Molecules on Acid or Spermidine Concentration. Anal. Sci. 25, 293–299 (2009). 19212068 10.2116/analsci.25.293

[b41] Yoshikawa K. & Matsuzawa Y. Discrete Phase Transition of Giant DNA Dynamics of Globule Formation from a Single Molecular Chain. Phys. D 84, 220–227 (1995).

