# Supplementary material for: Fluidic Grooves on Doped-Ice Surface as Size-Tunable Channels
Source: Sci Rep. 2015 Nov 25;5:17308. doi: 10.1038/srep17308 (PMC4658556; doi:10.1038/srep17308)
Supplement: Supplementary Information [file srep17308-s1.pdf]

Supplementary Information

Fluidic Grooves on Doped-Ice Surface as Size-Tunable Channels

Arinori Inagawa, Makoto Harada, and Tetsuo Okada*

Department of Chemistry, Tokyo Institute of Technology, Meguro-ku, Tokyo 152-8551, Japan

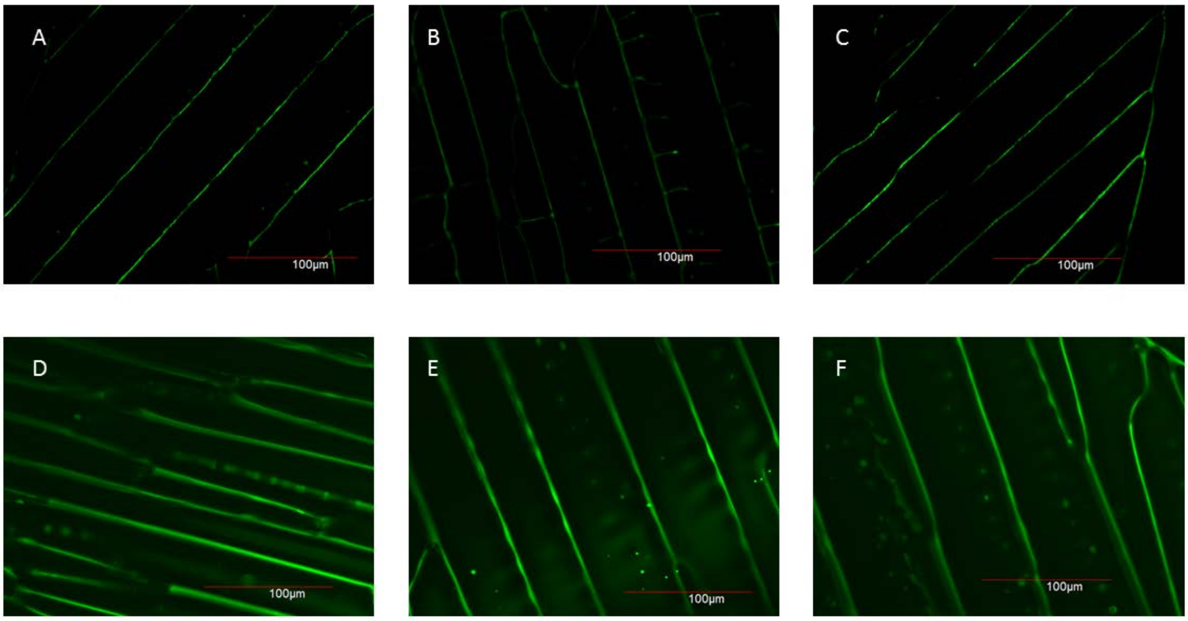

Figure S1 Morphologies of IGBs on the surfaces of sucrose-doped ice.

All of the frozen platforms were prepared at -6.0 °C and then the temperature was changed for observation. A-C: 75 mM sucrose at -12.0 °C. D-F: 100 mM sucrose at -8.0 °C. All of the images were taken for different ice preparation.

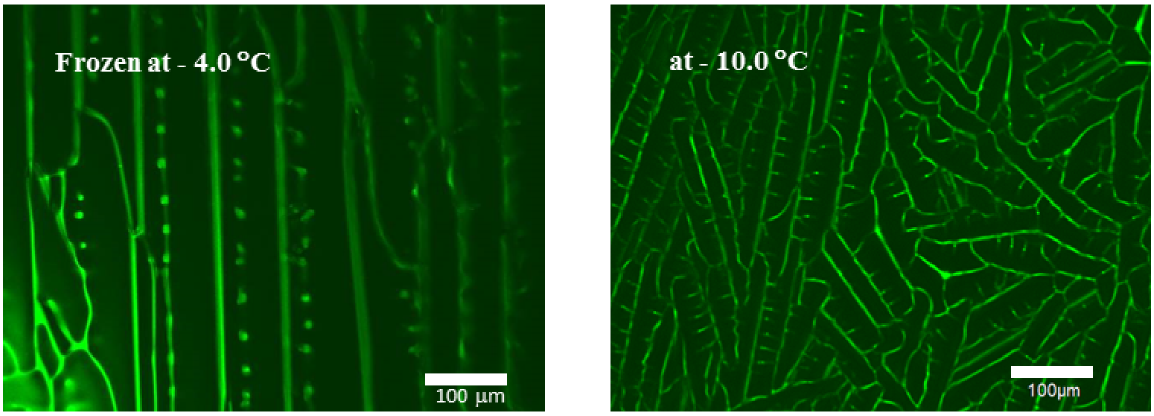

Figure 2 IGB morphologies of frozen samples prepared at different temperatures

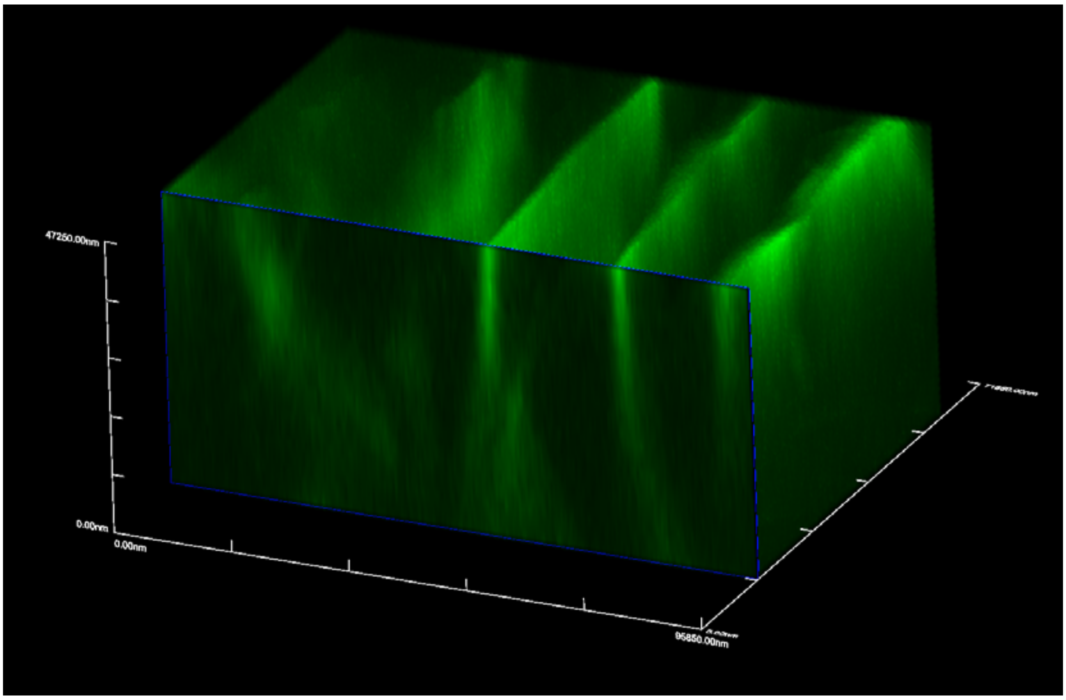

Figure S3 Three dimensional image of IGBs near the surface of 75 mM sucrose-doped ice at -12.0 °C.

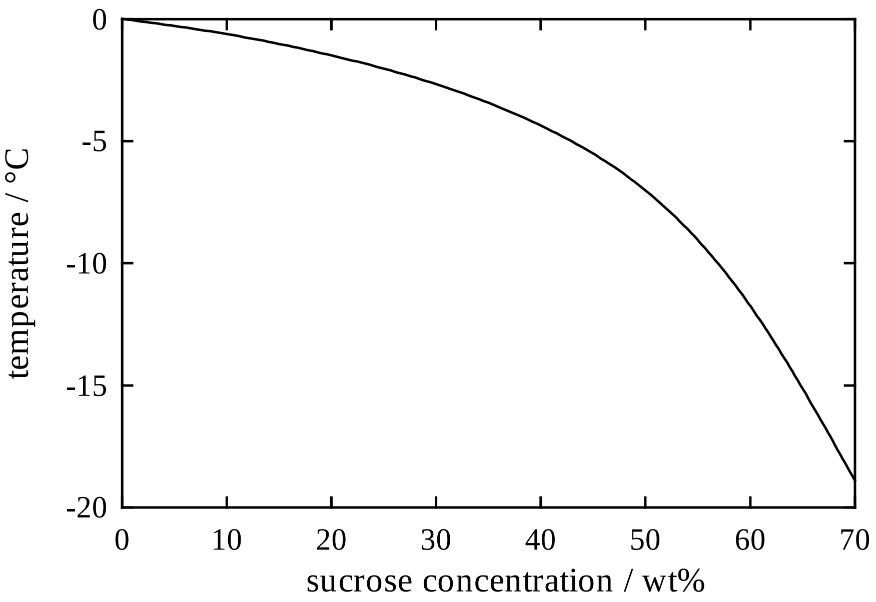

Figure S4 Phase diagram (freezing depression curve) for sucrose-water. Data taken from ref. 34.

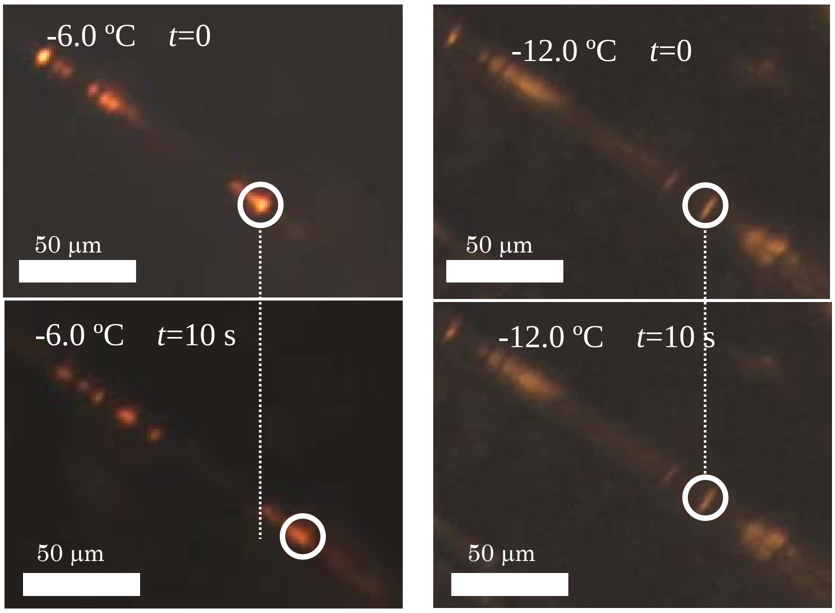

Figure S5 Temperature dependence of the electrophoretic migration of *d*=1.3 μm particles in the IGB channels. The particle migration is seen at -6 °C in the IGB channel prepared with 75 mM sucrose, whereas the particle became immobile at -12 °C in the same IGB channel.

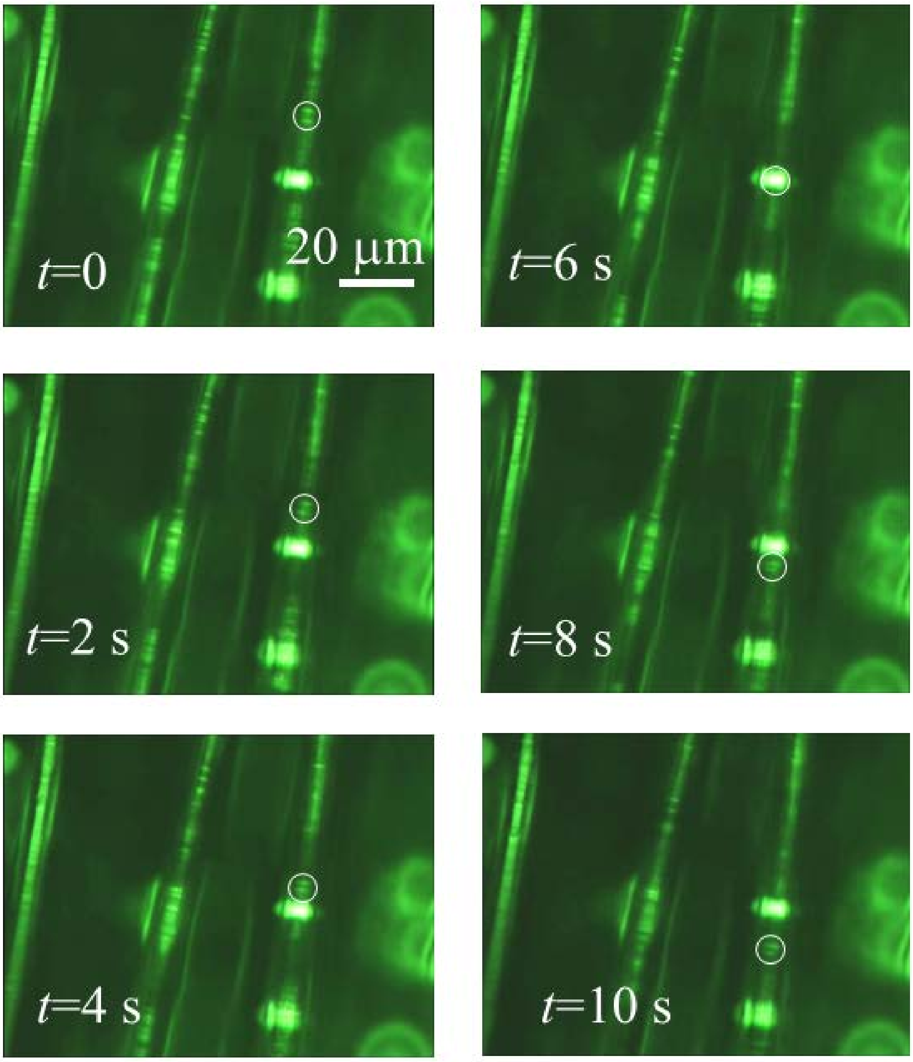

Figure S6 Particle migration through the spaces below or above already entrapped particle

The circle in each image shows migrating d=0.59 μm particle, which passes through entrapped d=3.8 μm particle.

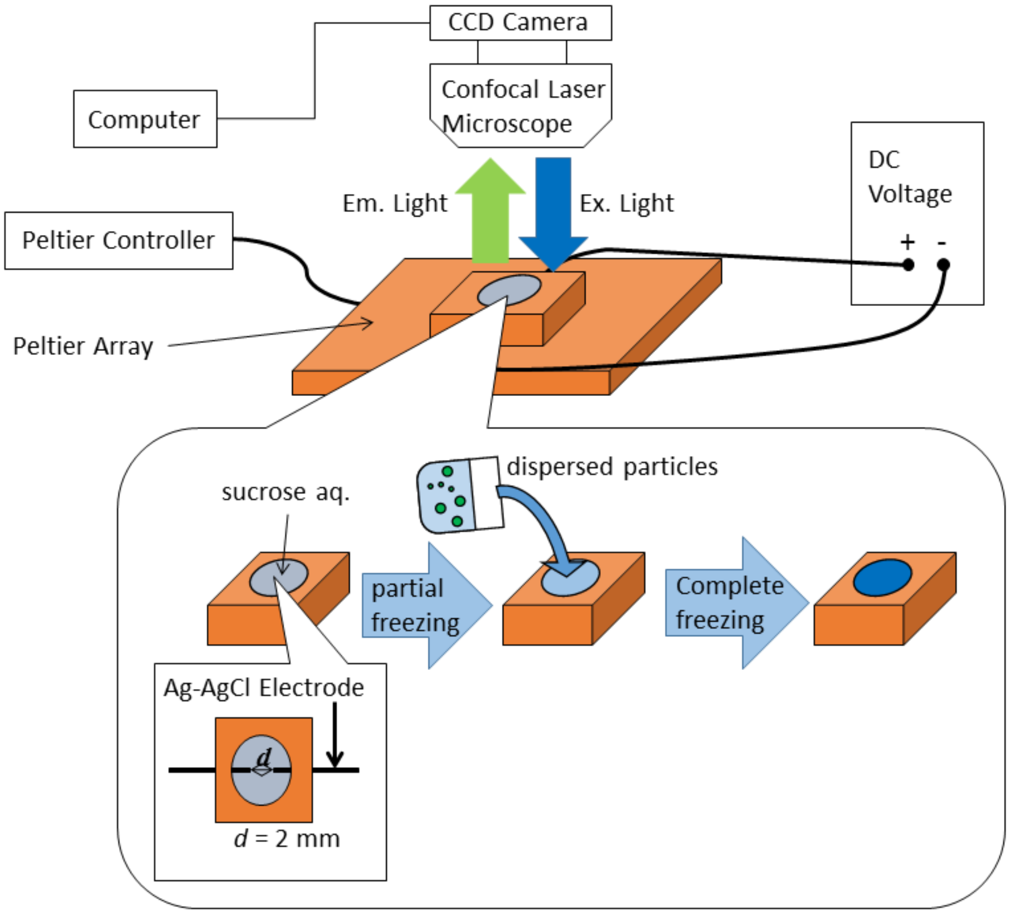

Figure S7 Schematic representation of experimental set-up

Table S1 Conductivity of the LP with predicted composition and size of the IGB channel

temperature / ºC	conductivity μS/m	σ [a]	NaCl/mM	I /μA [b]	A / $10^7 m^2$ [c]	r /μm [d]
-2	247	2.6	7.97	5.5	7.1	948
-4	211	2.6	13	1.7	2.6	570
-6	150	2.1	16.5	1	2.1	519
-8	103	3.6	19.1	0.6	1.8	484
-10	73	3.8	21	0.2	0.87	333
-12	45	4.3	22.7	- [e]	-	-

a, standard deviation of conductivity (*n*=3)

b, current under an applied voltage of 63.1 V between the electrodes 2.0 mm apart

c, cross-sectional area

d, radius of circular cross-section

e, not measurable

Table S2 Viscosity of aqueous sucrose at various temperatures

Sucrose concentration / wt%	η/ mPa s 0 ºC	10 ºC	20 ºC	30 ºC	40 ºC	50 ºC	60 ºC	70 ºC	80 ºC	90 ºC	100 ºC
10	2.49	1.77	1.38	1.06	0.85	0.71	0.6	0.51	0.45	0.39	0.35
20	3.8	2.65	1.96	1.5	1.19	0.97	0.81	0.69	0.59	0.51	0.46
30	6.6	4.5	3.17	2.35	1.84	1.48	0.2	1	0.85	0.73	0.63
40	14.8	9.8	6.2	4.38	3.25	2.5	1.98	1.61	1.33	1.12	0.96
50	45	26	15.5	10	7.3	5.1	3.93	3.05	2.5	2.01	1.38
60	238	110	56.9	33.5	21	13.9	9.6	7.1	5.5	4.2	3.3
70			460	214	111	63.1	38.8	24.8	16.7		
80					2160	830	394	204	115	83	

Data taken from “Kagaku Binran (Chemical Index)”, Ed. The Chemical Society of Japan,

Maruzen: Tokyo, 4th Edition, 1993.

Determination of the parameters in Eq.(2)

The sucrose concentration in the LP (c_{suc}^{LP}) at a given temperature was determined based on the water/sucrose phase diagram (Figure S4). The viscosities of this concentration (c_{suc}^{LP}) in a temperature range of 0-100 °C were determined by interpolating the relevant data listed in Table S2. The parameters in Eq.(2) were determined by curve-fitting of these viscosity data for given c_{suc}^{LP}

$$\eta = A\exp\left(\frac{-\Delta E}{RT}\right) \tag{2}$$

The parameters determined in this way are summarized in Table S3. These parameters allow us to predict the viscosities of the LP at the equilibrium temperature under frozen conditions. The parameters in the first line in Table S3 are, for example, determined for c_{suc}^{LP}=25.1 wt%. This concentration corresponds to that of LP at -2.0 ºC. Eq.(2) with these parameters predicts the viscosity of the LP at this temperature as shown in Table S4. Similar calculations were carried out for different temperatures.

Table S3 Parameters used in Eq.(2)

sucrose concentration [a] / wt%	ln A	ΔE / kJ mol^{-1}
25.1	12.8	16.7
37.9	12.9	19.2
46.4	14.2	24.1
52.8	15.6	29.0
57.1	15.1	29.4
60.8	15.0	29.9

a, The concentrations in this column correspond to the equilibrium concentration of sucrose in the LP (c_{suc}^{LP}) temperature at -2.0, -4.0, -6.0, -8.0, -10.0 and -12.0 ºC from the top to the bottom.

Table S4 Viscosity of the LP predicted with eq.(2) and data listed in Table S2

temperature / ºC	sucrose concentration / wt %	η / mPa s [a]
-2.0	25.1	4.59
-4.0	37.9	12.71
- 6.0	46.4	33.20
- 8.0	52.8	88.23
- 10.0	57.1	190.6
- 12.0	60.8	304.4

a, calculated from the values determined at higher temperatures based on Eq.2
